# Loss of spinal substance P pain transmission under the condition of LPA_1 _receptor-mediated neuropathic pain

**DOI:** 10.1186/1744-8069-2-25

**Published:** 2006-08-16

**Authors:** Makoto Inoue, Asuka Yamaguchi, Megumi Kawakami, Jerold Chun, Hiroshi Ueda

**Affiliations:** 1 Division of Molecular Pharmacology and Neuroscience, Nagasaki University Graduate School of Biomedical Sciences, 1-14 Bunkyo-machi, Nagasaki 852-8521, Japan; 2 Department of Molecular Biology, Helen L. Dorris Child and Adolescent Neuropsychiatric Disorder Institute, The Scripps Research Institute, 10550 N. Torrey Pines Road, La Jolla, CA 92037, USA

## Abstract

Among various machineries occurring in the experimental neuropathic pain model, there exists the loss of pain transmission through C-fiber neurons as well as the hypersensitivity through A-fibers. The current study reveals that molecular machineries underlying the latter hypersensitivity are derived from the events through LPA_1 _receptor and its downstream RhoA-activation following peripheral nerve injury. The loss of C-fiber responses, which are mediated by spinal substance P (SP) pain transmission was observed with the nociceptive flexor responses by intraplantar injection of SP in nerve-injured mice. The immunohistochemistry revealed that SP signal in the dorsal horn was markedly reduced in such mice. All these changes were completely abolished in LPA_1_^-/- ^mice or by the pretreatment with BoNT/C3, a RhoA inhibitor. In addition, the loss of C-fiber responses and the down-regulation of spinal SP signal induced by single intrathecal LPA injection were also abolished in such treatments. All these results suggest that the loss of pain transmission through polymodal C-fiber neurons is also mediated by the LPA_1 _activation following nerve injury.

## Findings

Injury to peripheral nerves cause rearrangement of synaptic contacts, wind up, central sensitization, long-term potentiation and loss of inhibitory neuron of the spinal dorsal horn [1-7]. Retraction and the following lack of signals from polymodal C-fiber terminals to dendritic targets in lamina II of the dorsal spinal cord, and sprouting of mechanoreceptor Aβ-fibers (which terminate in laminae III-V) to form new connections with the vacated dendrites of the second order nociceptive neurons in lamina II, are suspected to be partially responsible for the abnormal pain sensations, or allodynia associated with peripheral nerve damage [2,5,8]. The mechanisms behind these rerouting processes may be driven by specific factors mediating neurite retraction.

The bioactive lipid mediator, lysophosphatidic acid (LPA), which is generated after nerve injury and implicated in pathological pain, signals through the LPA_1 _receptor to activate G_12/13 _and the small GTPase signaling molecule RhoA, may serve as a candidate signaling-molecule for inducing the retraction of polymodal C-fibers *in vivo *as it has been found to induce neurite retraction *in vitro *[9-15]. Recently, we reported that the LPA_1 _receptor is necessary to initiate neuropathic pain, neuronal demyelination and up-regulation of pain-related proteins following nerve injury [16]. In addition, intrathecal (i.t.) injection of LPA mimics nerve injury-induced behavioral, morphological and biochemical changes[16]. Inhibition of the RhoA downstream pathway of LPA_1 _receptor activation has shown that this pathway is essential for both nerve injury- and LPA-induced neuropathic pain.

In the present study, we sought to determine, whether activation of LPA_1 _receptor and the downstream RhoA pathway are required for abrogation of polymodal C-fiber signaling following nerve injury. Decreased polymodal C-fiber signaling was evaluated by spinal SP immunohistology as well as nociceptive responses through SP-containing polymodal C-fibers following nerve injury. Furthermore, we investigated whether i.t injection of LPA would mimic the spinal SP and nociceptive profile of mice subjected to nerve injury.

Male Std-ddY mice, and mice lacking the *lpa*_1 _gene (LPA_1_^-/-^), and its wild typed mice weighing 20–22 g maintained at 21 ± 2°C with food and water ad libitum were used throughout the experiments. All procedures were approved by the Nagasaki University Animal Care Committee and complied with the recommendations of the IASP [17]. Partial ligation of the sciatic nerve was performed as described previously [16,18]. Experiments were carried out at 7 days. Algogenic-induced paw-flexor response (APF) tests were performed as described previously [6,19]. LPA (Avanti Polar-Lipids, Alabaster, AL) was dissolved in A-CSF (NaCl 125 mM, KCl 3.8 mM, CaCl_2 _2.0 mM, MgCl_2 _1.0 mM, KH_2_PO_4 _1.2 mM, NaHCO_3 _26 mM, Glucose 10 mM), and was injected intrathecally (i.t.) between lumbar 5 and 6 regions. Most of the experiments were conducted 24 h following injection LPA (1 nmol in 5 μl). BoNT/C3 was a kind gift from Dr. Shuji Kozaki. BoNT/C3 was delivered intrathecally (10 pg in 5 μl) 1 hr prior to nerve-injury or LPA-treatment. All behavioral experiments were carried out by investigators blinded to the drug-treatment.

For histological examinations, mice were deeply anesthetized and perfused with potassium-free phosphate-buffered saline (K^+ ^free PBS, pH7.4), followed by 4% paraformaldehyde. The spinal cord was removed, post-fixed for 3 hours and cryoprotected overnight in 25% sucrose and finally fast frozen in cryoembedding compound. The spinal cord was cut at 30 μm and incubated with 50% methanol for 10 min, and then 100% methanol for 10 min. The sections were washed, incubated with blocking buffer containing 2% BSA for 60 min, and then reacted overnight at 4°C with goat anti SP antibodies (1:500) in blocking buffer. After washing, the sections were placed in Alexa488-conjugated secondary antibodies (1:300) for 120 min at RT. After washing, spinal cord sections were mounted on glass slides, treated with PermaFluor (Thermo Shandon, Pittsburgh, PA), protected with cover slips and examined by fluorescence microscopy (Keyence, Japan). Changes in spinal cord SP immunoreactivity were analyzed with the Image J software on digital images with a grey scale from 0 to 255. The SP signal was determined as the total signal in the superficial dorsal horn after background subtraction. Statistical analyses were performed using Student's *t-*test. Significance was set to p < 0.05. All results are expressed as means ± S.E.M.

After the confirmation that mice showed no spontaneous moving approximately 20 min after the cannulation, they were intraplantarly (i.pl.) given with SP (Peptide Institute, Osaka, Japan) to see changes in nociceptive flexor responses by nerve injury or LPA-treatment. SP induced a dose-dependent nociceptive flexor response from 0.01 to 1 pmol in sham-operated mice, but not in injured mice (Table 1) as previously reported in Std-ddY mice[6]. However, the loss of SP-sensitivity in injured mice was reversed in LPA_1_^-/- ^mice (Table 1). Similar reversal was also observed in mice i.t. pretreated with 10 pg of C3-typed botulinum toxin (BoNT/C3), a selective inhibitor of ADP-ribosylation of RhoA (Table 1). The loss of SP-sensitivity was also observed when mice were treated with LPA (1 nmol, i.t.) 24 hr prior to the APF test (Table 1). Similarly, the loss of function was also reversed in LPA1^-/- ^mice or in mice pretreated with BoNT/C3 (Table 1).

**Table 1:** Loss of nerve injury- and LPA-induced spinal SP-mediated functional reduction in LPA_1_^-/- ^mice and mice with BoNT/C3 treatment.

	SP (0.01 pmol, i.pl.)	SP (0.1 pmol, i.pl.)	SP (1 pmol, i.pl.)
WT (Sham)	19.2 ± 2.4	36.0 ± 1.9	52.8 ± 2.4
WT (Injury)	5.7 ± 3.6 *	10.9 ± 3.9 *	8.6 ± 6.6 *
KO (Injury)	20.0 ± 4.5 #	34.9 ± 4.6 #	52.2 ± 2.1 #
Sham (Veh)	13.0 ± 2.3	23.8 ± 3.1	66.2 ± 6.6
Injury (Veh)	3.8 ± 0.5 *	6.1 ± 3.7 *	14.6 ± 7.7 *
Injury (BoNT/C3)	11.1 ± 6.8 #	21.2 ± 5.6 #	65.1 ± 8.8 #
WT (Veh)	10.1 ± 3.6	23.9 ± 4.6	53.2 ± 3.2
WT (LPA)	0 *	6.3 ± 0.8 *	11.1 ± 4.0 *
KO (LPA)	13.3 ± 2.6 #	28.2 ± 2.0 #	52.9 ± 1.7 #
Veh (Veh)	9.2 ± 1.2	21.6 ± 3.2	64.8 ± 6.0
LPA (Veh)	1.2 ± 0.2 *	4.8 ± 0.1 *	12.0 ± 4.8 *
LPA (BoNT/C3)	10.1 ± 1.9 #	19.2 ± 4.8 #	62.4 ± 6.0 #

Nociceptive flexor responses induced by injection (2 μl) of SP at each dose were normalized to the 'maximal reflex' defined as the biggest response among the responses occurring immediately after cannulation. Significance was set to *, #*p *< 0.05. Data are presented as means ± S.E.M. from experiments using at least 6 mice.

WT (Sham), WT (Injury) and KO (Injury) represent LPA_1_^+/+ ^mice with sham-operation, LPA_1_^+/+ ^mice with injury, and LPA_1_^-/- ^mice with injury, respectively.

Sham (Veh), Injury (Veh) and Injury (BoNT/C3) represent sham-operated mice with vehicle treatment, injured mice with vehicle treatment, and injured mice with BoNT/C3 (10 ng, 1 hr before injury), respectively.

WT (Veh), WT (LPA) and KO (LPA) represent LPA_1_^+/+ ^mice with vehicle-treatment, LPA_1_^+/+ ^mice with LPA-treatment injury, and LPA_1_^-/- ^mice with LPA-treatment, respectively.

Veh (Veh), LPA (Veh) and LPA (BoNT/C3) represent vehicle-treated mice with vehicle treatment, LPA-treated mice with vehicle treatment, and LPA-treated mice with BoNTC3 (10 ng, 1 hr before LPA treatment), respectively.

**Figure 1:**
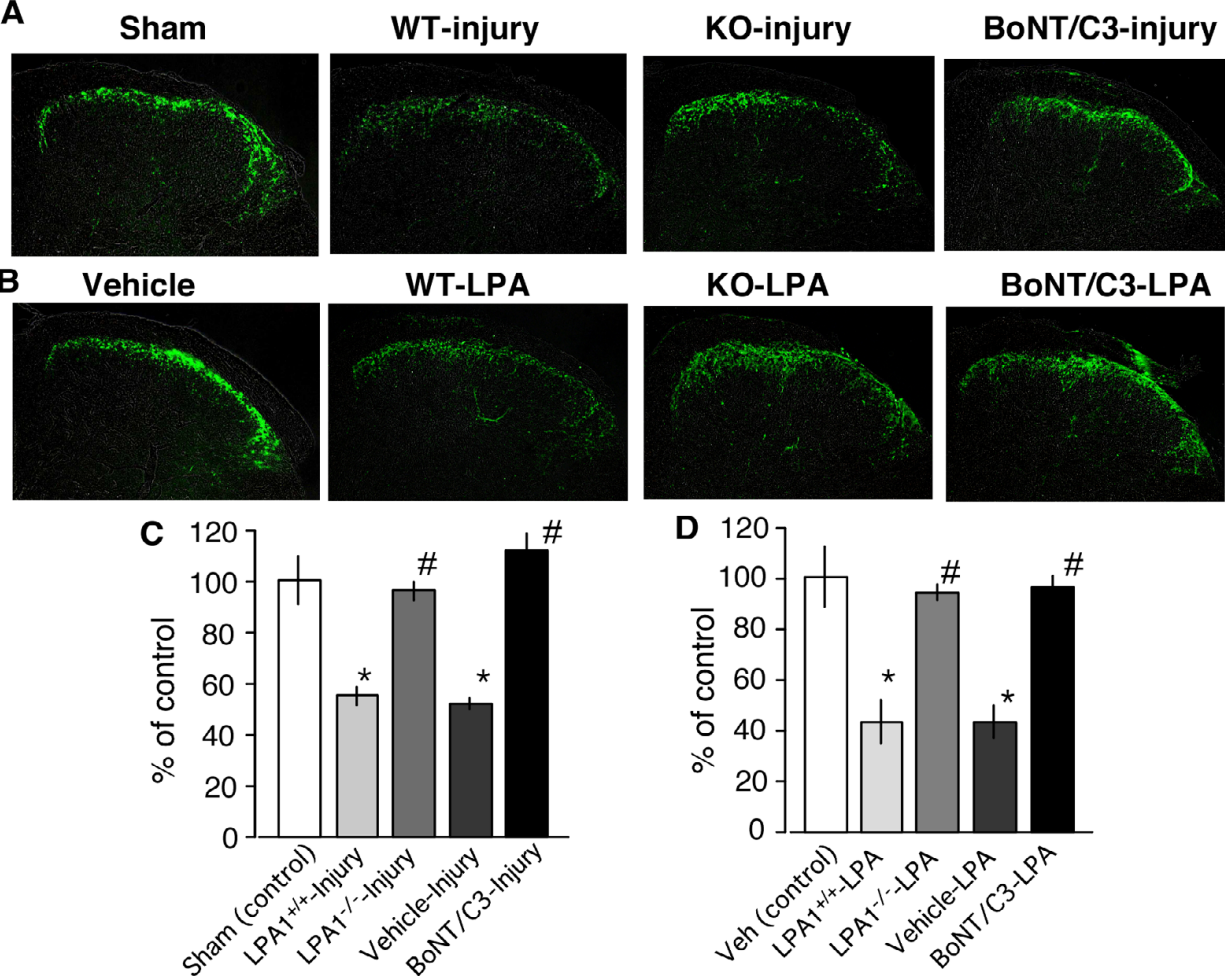
**Lack of nerve injury- and LPA-induced reduction of spinal SP immunoreactivity in LPA_1_^-/- ^mice and mice with BoNT/C3 treatment**. (A) Loss of nerve injury-induced reduction of SP immunoreacctivity in LPA_1_^-/- ^mice and mice with BoNT/C3 pretreatment (10 pg, 1 hr prior to injury). (B) Loss of LPA (1 nmol, i.t.)-induced reduction of SP immunoreacctivity in LPA_1_^-/- ^mice and mice with BoNT/C3 pretreatment. (C, D) Quantification of SP immunoreactivity in nerve injured mice (C) or in LPA-treated mice (D). Data are presented as means ± S.E.M. from experiments using at least 3 mice.

As shown in Fig. 1A, a marked decrease in SP immunoreactivity in the ipsilateral superficial dorsal horn was observed at day 7 in nerve injured mice compared to sham operated controls, as previously reported[20]. The SP immunoreactivity in the lamina I and II of spinal dorsal horn was significantly reduced to approximately 50% of control by the nerve injury (Fig. 1C). The injury-induced reduction was completely prevented in LPA1^-/- ^mice and in mice pretreated with BoNT/C3 (10 pg, i.t.) 1 hour prior to the injury (Fig. 1AC). Quite similar results were also observed when LPA was applied 24 h prior to the observation (Fig. 1BD).

We have recently proposed that nociceptive fibers could be pharmacologically classified into three types in terms of neonatal capsaicin-sensitivity and spinal antagonism, by use of APF test in mice[6]. This classification would have an important advantage that nociceptive responses through three different fibers were distinctly affected in mice with partial sciatic nerve injury. There is a loss of nociceptive responses through type-1 C-fibers, which are sensitive to neonatal capsaicin, and use SP and NK1 receptor as the spinal pain transmission. Although no change is found in the nociceptive responses through type-2 C-fibers, which are also sensitive to neonatal capsaicin, but use glutamate and NMDA receptor, marked enhancement is observed with the nociceptive responses through type-3 A-fibers, which are not sensitive to neonatal capsaicin, but use glutamate and NMDA receptor. As shown in Table 1, the nociceptive response induced by SP (i.pl.) through type-1 C-fibers is the case, which showed a complete loss of responses in mice with injury. Similar results were observed with B2-type bradykinin receptor agonist and nociceptin/orphanin FQ [18,21].

The major conclusion of the present study is that LPA_1 _receptor activation is also involved in this loss of function through type-1C fibers in mice with nerve injury, which causes neuropathic pain and underlying alterations of gene expressions and demyelination. BoNT/C3 is a useful pharmacological tool to block this LPA_1_-mediated changes, since it shows a potent and long-lasting blockade of RhoA pathway, a major downstream of signaling[16]. Another excellent tool is LPA_1_^-/- ^mice, which abolish the nerve injury-induced thermal hyperalgesia and mechanical allodynia[16]. In experiments using these two tools, nerve injury-induced loss of SP (i.pl.)-mediated C-fiber responses are mediated through RhoA activation through LPA_1 _receptor. The use of pharmacological neuropathic pain model following single i.t. injection of LPA also provide the important evidence for the validity of this conclusion.

Such a loss of type-1 C-fiber responses would be explained by the finding that spinal SP-immunoreactivity was markedly reduced in mice with injury (Fig. 1A). The use of BoNT/C3, LPA_1_^-/- ^mice and LPA (i.t.)-induced neuropathic pain model revealed that down-regulation of SP-immunoreactivity in the dorsal horn is also mediated through the activation of LPA_1 _receptor and RhoA. These observations are in a good contrast with the previous findings that the nerve injury-induced activation of LPA_1 _receptor and RhoA causes functional changes in myelinated A-fibers, such as demyelination and up-regulation of Ca^2+ ^channel α2δ-1 subunit in medium/large neurons of DRG, both which may underlie the molecular mechanisms for neuropathic pain [16]. Regarding to the site of LPA action, we have observed that LPA (i.pl.) caused nociception through type-1 C-fiber [22,23], and demyelination of A-fibers [16]. However, it remains which A-fibers or myelinating Schwann cells are target underlying LPA-induced demyelination. The study using *ex vivo *culture experiments would be the next subject.

The present study demonstrates that the loss of spinal SP transmission via C-fibers is mediated through the activation of LPA_1 _receptor and RhoA, as seen in the case with up-regulation of A-fibers.

## Abbreviations

SP: substance P, LPA: Lysophoshatidic acid, BoNT/C3: C3-typed botulinum toxin, APF: Algogenic-induced paw-flexor response, i.t.: intrathecal, i.pl.: intraplantarly
